# A review of the bioactive components and pharmacological properties of *Lavandula* species

**DOI:** 10.1007/s00210-023-02392-x

**Published:** 2023-02-11

**Authors:** Gaber El-Saber Batiha, John Oluwafemi Teibo, Lamiaa Wasef, Hazem M. Shaheen, Ayomide Peter Akomolafe, Titilade Kehinde Ayandeyi Teibo, Hayder M. Al-kuraishy, Ali I. Al-Garbeeb, Athanasios Alexiou, Marios Papadakis

**Affiliations:** 1 Department of Pharmacology and Therapeutics, Faculty of Veterinary Medicine, Damanhour University, Damanhour, 22511 AlBeheira Egypt; 2 Department of Biochemistry and Immunology, Ribeirão Preto Medical School, University of São Paulo, Ribeirão Preto, São Paulo Brazil; 3 Department of Biochemistry, University of Ibadan, Ibadan, Nigeria; 4 Department of Maternal-Infant and Public Health Nursing, College of Nursing, Ribeirão Preto, University of São Paulo, Ribeirão Preto, São Paulo Brazil; 5 Department of Pharmacology, Toxicology and Medicine, Medical Faculty, College of Medicine, Al-Mustansiriyah University, P.O. Box 14132, Baghdad, Iraq; 6 Department of Science and Engineering, Novel Global Community Educational Foundation, Hebersham, NSW 2770 Australia; 7 AFNP Med, 1030 Vienna, Austria; 8 Department of Surgery II, University Hospital Witten-Herdecke, Heusnerstrasse 40, University of Witten-Herdecke, 42283 Wuppertal, Germany

**Keywords:** *Lavandula* species, Medicinal properties, Volatile/essential oil, Antimicrobial, Anti-diabetic, Neurological properties

## Abstract

*Lavandula* species is a flowering plant that is common in Europe and across the Mediterranean. Lavender has many health benefits for humans. In addition to its use in herbal medicine, it is widely used in the fields of cosmetics, perfumes, foods, and aromatherapy. Google Scholar, PubMed, Scopus, and Web of Science were used to search for relevant material on the phytochemical ingredients, the pharmacologic effects of the ingredients, and the mechanism of action of the *Lavandula* species identified. These materials were reviewed in order to have access to important updates about the *Lavandula* species. Lavender as referred to in English contains essential oils, anthocyanins, phytosterols, sugars, minerals, coumaric acid, glycolic acid, valeric acid, ursolic acid, herniarins, coumarins, and tannins. It has been used to treat colic and chest ailments, worrisome headaches, and biliousness, and in cleaning wounds. It has antifungal, antibacterial, neurologic, antimicrobial, anti-parasitic, anti-diabetic, and analgesic effects among others. *Lavandula* species has prospects for various biological applications, especially with its dermatological application. Advances in drug development would enable characterization of various bioactive constituents; thus, its development and application can have a more positive impact on humanity. Here, we highlighted updated information on the history, distribution, traditional uses, phytochemical components, pharmacology, and various biological activities of *Lavandula* species.

## Introduction

Among the plants of the Labiatae (Lamiaceae) family, lavender (*Lavandula* sp.) is known for its medicinal properties (Simpson 2019). The genus *Lavandula* is indigenous to the regions bordering the Mediterranean Sea, from southern Europe through north and east Africa, the Middle East, southwest Asia, and southeast India. Hundreds of hybrids, dozens of subspecies, and more than 30 species are known (Koulivand et al. 2013). *L. angustifolia*, generally referred to as English lavender, is one of the four main species of lavender. It is a frost-resistant species with a variety of attractive cultivars and a beautiful flower color (formerly known *as L. vera* or *L. officinalis*). Other species are *L. latifolia*, a Mediterranean grass-like lavender; *L. stoechas*, a huge plant with greenish-gray foliage and late blooming with a potent scent (also known as French lavender); and *L. intermedia*, a sterile hybrid between *L. latifolia* and *L. angustifolia* (Cavanagh and Wilkinson 2002a). There are several health benefits that lavender has for body functions. Lavender is widely utilized in the aromatherapy, food, cosmetics, and perfume sectors in addition to herbal medicine (Prusinowska and ´Smigielski K. 2014). Volatile oils (linalool), limonene, perillyl alcohol, linalyl acetate, cis-smine, terpene, coumarin, tannin, caffeic acid, and camphor are considered to be the major constituents of lavender. However, various species have varying relative concentrations of each of these chemicals (Bikmoradi et al. 2017). Linalool affects the central nervous system’s aminobutyric acid receptors to produce sedation (Rai et al. 2020). According to certain studies on the benefits of lavender, including systematic reviews and meta-analyses, the herb greatly lessens labor pain (Kazeminia et al. 2020; Mirzaiinajmabadi et al. 2018; Makvandi et al. 2016), dysmenorrhea (Mousavi Kani et al. 2019), episiotomy healing (Abedian et al. 2020), depression (Firoozeei et al. 2021), the quality and treatment of sleep disorders (Mameneh et al. 2021; Fismer and Pilkington 2012; Lin et al. 2019), and blood sugar levels (Sebai et al. 2013). In addition to being used in bathing, lavender is also employed in aromatherapy and massage (Koulivand et al. 2013). Because it has few adverse effects, aromatherapy—the therapeutic inhalation of essential oils—is a popular method of reducing stress (Bikmoradi et al. 2017).

### Origins and cultivation (Fig. 1)

**Fig. 1:**
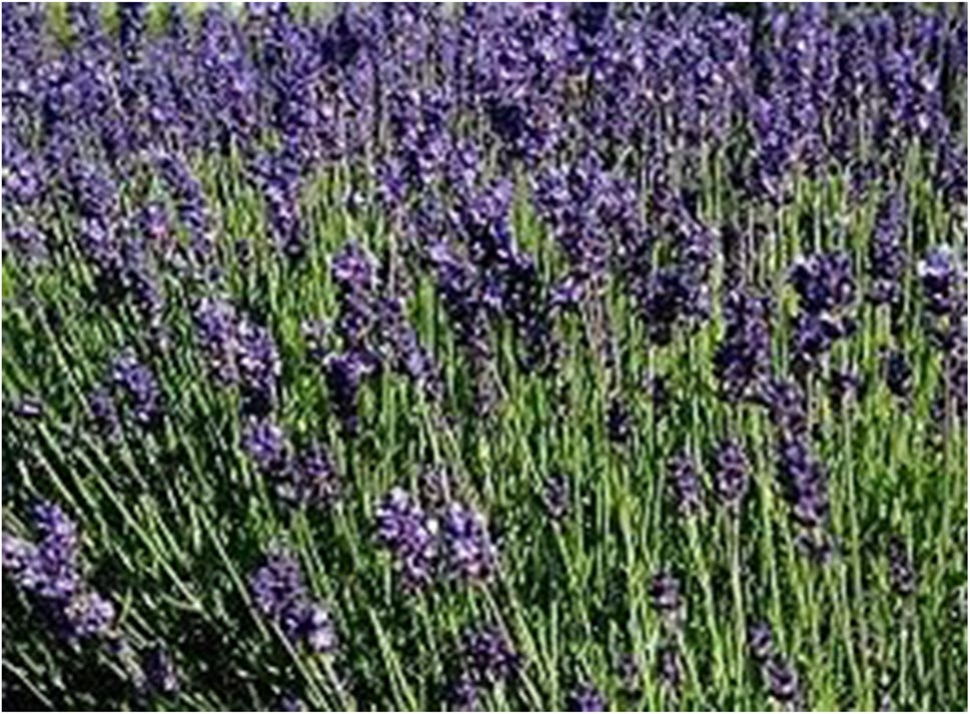
Lavandula species

Lavender (*Lavandula angustifolia*, *Lavandula officinalis*, *Lavandula vera*) is a perennial evergreen plant that is also known as therapeutic lavender, true lavender, and common lavender. Lavender is a Mediterranean plant native to France, Spain, Andorra, and Italy, although it is also grown in Poland (Boelens 1995; Smigielski et al. 2009). The Latin verbs “lavo” and “lavare” mean “wash” or “clean” and give the name lavender. The various common names of lavender and its related substances include common lavender, garden lavender, English lavender, *Lavandula burnamii*,* Lavandula dentate*, *Lavandula dhofarensis*, *Lavandula latifolia*, *Lavandula officinalis* L., *Lavandula stoechas*, limonene, perillyl alcohol, pink lavender, perillyl alcohol, true lavender, and white lavender (Basch et al. 2004). Lavender has been used medicinally for a long time, according to the Dioscorides publication De Materia Medica, which celebrates the medicinal properties of herbs. The Romans used lavender as a bath additive and in the Middle Ages it was one of the most valuable essential oil plants used to make perfumes and soaps. It is used both as a laxative and as a food additive. Lavender grows in a dense, homogeneous mass up to a height of 40–60 cm. The bottom of the trunk is a tree and the upper half is green. Lavender has linear or lanceolate leaves with wavy edges and a highly branched fibrous root system. Tomentum protects silver-green lavender leaves from direct sunlight, wind, and excessive moisture loss by covering them with a thick layer of felt. Lavender flowers form a ring on the top of the stem and bloom in spikes (3–5 flowers per ring). Despite the development of white (Alba and Nana Alba) and pink (Rosea) varieties, they have a pale purple color (Góra et al. 2005). Lavender is well-drained and grows best on fertile, calcareous soils (*Lavandula angustifolia*). It thrives in full sun with some wind protection. Few years after cultivation, lavender can be enriched with organic manure or artificial fertilizers. This promotes excessive green growth and reduction of shoot sites. In Poland, lavender is not completely frost-resistant and requires a thick winter cover (Prusinowska and Śmigielski 2014). Plants can be grown vegetatively, tissue cultured, or generatively from seeds, as well as soft and hard wood cuttings. Pruning lavender shrubs on a regular basis increase flowering and plant growth. From July to August, the plant is in full flower. Harvesting is best done on a sunny, dry day. Before opening, flowers should be picked and dried in bundles in a cool, well-ventilated location. Flowers (*Flos Lavandulae*) and flowering aerial parts (*Herba Lavandulae*) are used for herbal purposes, and essential oils are made from fresh or dried tops of flowering plants (Smigielski et al. 2009).

## Mechanism of action

Linalool, perillyl alcohol, linalyl acetate, camphor, limonene, tannin, triterpene, coumarin, cineole, and flavonoids are one of the more than 100 ingredients of lavender. Linalool has been found to suppress motor activity in mice by dose-dependent binding to glutamate, an important excitatory neurotransmitter in the central nervous system, and it is hypothesized that GABA enhancement is responsible for the hypnotic and anticonvulsant properties of lavender (Elisabetsky et al. 1995). The mechanism underlying lavender’s spasmolytic effects is unknown. In vitro, Gamez et al. investigated the anti-plasmodic effect of *L. dentate* (a type of lavender). Acetylcholine-induced muscle contraction has been shown to be significantly suppressed by cineole. Cineol was found to have a strong inhibitory effect on acetylcholine-stimulated muscle contractions. According to Lis Balchin et al. (Lis-Balchin and Hart 1999), linalool and linalyl acetate in *L. angustifolia* oil can cause cAMP-mediated relaxation of guinea pig ileum smooth muscle. The physiological effects of lavender on the sympathetic nervous system activity are attributed to a cAMP-based mechanism, according to the study. According to Fulton et al., perillyl alcohol (POH) triggers cell proliferation in smooth muscle cell cultures (Ren and Gould 1994).

In rats, both limonene and POH have been shown to inhibit tumor growth and stimulate apoptosis (Mills et al. 1995; Haag and Gould 1994). According to an in vitro study of the effects of POH on the development of lung cancer, POH prevented farnesylation, the step that activates the oncogene Kras (Lantry et al. 1997). The lipid-lowering effect of lavender is associated with cineole, a cyclic monoterpene that lowers cholesterol in rats by blocking the HMGCoA enzyme (Clegg et al. 1982). Perillyl alcohol (POH), a component of lavender, has been shown to prevent the conversion of lathesterol to cholesterol (Ren and Gould 1994). Caffeic acid in lavender has been shown to have antioxidant properties in vitro (Hohmann et al. 1999).

### Properties and application

Lavender (*L. angustifolia*) flowers, buds, and leaves are edible and are used to flavor broth and jelly (not consumed as a raw material). The aroma of lavender is great at repelling moths and flies; thus, the plant is kept in closets and drawers. This insecticidal effect has been demonstrated in various research (Perrucci et al. 1994; O’Brien 1999). Injections and tinctures made from lavender flowers have calming and pain-relieving properties. One study (Akhondzadeh et al. 2003) found that lavender tincture helps with depression, migraine, and anxiety. In mice, lavender extract reduced dementia associated with Alzheimer’s disease (Kashani et al. 2011) and stopped the growth of carcinogenic cells in cytotoxicity studies of the extract’s effects on lung cancer (Shen et al. 2009). Perfumes, cosmetics, and household chemicals all contain lavender essential oils. Toilet water, cologne, lotions, and aftershave all have unique top notes, and household cleaning products have a fresh, clean scent. Lavender-scented products are available from Avon, Procter and Gambling, and Aloe Vera. It is just one example of a famous cosmetic brand. Lavender (*L. angustifolia*) essential oil is bactericidal at doses of 4.0–9.0 mg/mL (Mayaud et al. 2008).

Lavender plants produce essential oils with a wide range of biological activities. *Salmonella*, *Enterobacter*, *Klebsiella*, *E. coli*, *Listeria monocytogenes*, and *Listeria monocytogenes* are all inhibited by *Lavandula dentata* essential oil. Essential oil of *L. bipinnata* contains an antibacterial agent (against *E. coli*, *P. aeruginosa*, *S. aureus*, *B. subtilis*) and antifungal agent (*A. niger*, *P. notatum*, and *C. albicans*) capability at doses of 0.5–2.0 gml^−1^ for bacteria and 2.0–4.0 gml^−1^ for fungi (Hanamanthagouda et al. 2010). At a concentration of 0.6 or 1.0 lml^−1^, lavender essential oil has high inhibitory property against the growth of the gram-positive bacteria (*Bacillus subtilis*, *S. aureus*) and gram-negative bacteria (*Escherichia coli*, *Pseudomonas aeruginosa*) (Prusinowska, unpublished). In Poland, the antibacterial activity of lavender essential oil (*L. angustifolia*) has been studied. The essential oil has been reported to be active against yeasts and molds like *Candida* sp., *A. niger*, and *P. expansum*, with MICs 2.5–3 times lower than bacteria. In addition to *E. coli*, *P. aeruginosa*, *S. aureus*, *B. subtilis*, *Candida* sp., and *A. niger*, lavender hydrolates have antimicrobial activity against *E. coli*, *P. aeruginosa*, *S. aureus*, *B. subtilis*, *Candida* sp., and *A. niger* (Prusinowska, unpublished). The antioxidant properties of essential oils protect cells from damage from free radicals. According to Economou et al. (Economou et al. 1991), linoleic acid reduces fat oxidation and lipid peroxidation in the linoleic acid model system (Hui et al. 2010). Lin et al. (2009) utilized DPPH to investigate the antioxidant characteristics of lavender essential oil (*L. angustifolia*), most especially its ability to remove free radicals. At a concentration of 5gl^−1^, the value of 15.18 ± 0.009% suggests properties similar to those of lime and marjoram essential oils. Viuda Martos et al. (Viuda-Martos et al. 2011) found that the potential for essential oils to inactivate free radicals is significantly reduced by the same amount (4.11%) (Viuda-Martos et al. 2011). The ability of this essential oil to remove 50% DPPH radicals was tested with results ranging from 28.9 gml1 (Hussain et al. 2011) to 48.7 mgml1. This value was given as IC_50_ = 338.0 ml × ml^−1^ in a study of essential oils isolated from lavender grown in Poland (Prusinowska, unpublished). It was shown in study by Buchbauer et al. (Buchbauer et al. 1991) that some components of essential oils such as linalool and terpineol affect the central nervous system, reducing physical activity in humans and animals, reducing anxiety, helping sleep, and enhancing systemic effects in mice and rats. It shows that infusion of lavender essential oil causes drowsiness. After inhaling lavender essential oil, 40 healthy participants in one study showed greater EEG activity and improved on math tests. Patients report not only fatigue, but also relaxation and a positive outlook for life (Diego et al. 1998). Human experiments assessing the sleep-inducing properties of lavender oil have shown that the use of lavender oil aromatherapy increases sleep time and reduces drug use in patients suffering from hypnosis (Hudson 1995). A clinical trial of 245 people found that 72% of those who inhaled lavender oil slept soundly, compared to only 11% of those in the control group. In the treatment group, 4 out of 5 patients reported general well-being, compared with only 25% in the control group (Hudson 1995). According to a pig study, lavender (*L. angustifolia*) has anxiolytic effects. In animals, lavender-covered vehicle floors significantly reduced the incidence and severity of motion sickness (assessed by cortisol levels in saliva) (Bradshaw et al. 1998). Randomized clinical trials have shown that essential oil aromatherapy reduces anxiety in 122 critically ill patients compared to those who received massage or rest without aromatherapy. There was no significant difference in blood pressure or respiratory health between the two groups of subjects (Dunn et al. 1995). Lavender essential oil aromatherapy reduces anxiety associated with expected unpleasant symptoms, according to a study conducted in the dental care waiting room (Kritsidima et al. 2010a). According to Yamada et al. (1994), seizures caused by pentylenetetrazol or nicotine can be minimized by inhaling or injecting lavender essential oil into the abdominal cavity. This essential oil has been shown to have analgesic and anesthetic effects in vitro (Skoglund and Jorkjend 1991) and rabbit tests (Ghelardini et al. 1999). In HIV-positive adolescents, massage with lavender essential oils reduced the need for analgesics and, in certain situations, completely eliminated pain (Styles 1997). Rabbits inhaled lavender essential oil to reduce cholesterol and atherosclerosis of the aorta without affecting serum cholesterol levels (Nikolaevskiĭ et al. 1990). Studies (Romine et al. 1999) show that inhalation of lavender essential oil reduces systolic and diastolic blood pressure and heart rate. Lavender essential oils help treat gastrointestinal disorders by regulating gastrointestinal movements and bile ducts, reducing gas and bloating. In rats, inhalation of lavender essential oil has been shown to stimulate bile synthesis and restore normal catabolic oxidase activity (Gruncharov 1973). In guinea pigs, lavender essential oil was shown to be a smooth muscle relaxant that suppresses the contractile response of acetylcholine and histamine (Lis-Balchin and Hart 1997). Lavender is used to enhance hair growth and as an aphrodisiac. In a study of 31 men who sniffed 30 different odors, Hirsch and Gruss (1999) studied a group of 31 males who inhaled 30 various fragrances and discovered that lavender and pumpkin dough induced the greatest increase in blood flow to the penis—by 40% when compared to the control. It was discovered that the paste enhances treatment of sexual dysfunction. Eighty-six (86) patients with alopecia areata received a 7-week massage with essential oils containing lavender, and nearly half of the patients had improved hair growth (Hay et al. 1998). Lavender hydrolate has a relaxing effect and is also useful for insomnia and headaches. Both lavender hydrolates and fluidolates are effective in natural and organic cosmetics. Based on their chemical composition, fluidolates are believed to have similar properties, but more research is needed in this area. According to current literature reviews, lavender (*L. angustifolia*) and its phytochemicals have multi-directional bioactivity.

## Traditional uses

Antioxidants (Chrysargyris et al. 2016; Biswas et al. 2009), antifungal and bactericidal (Djenane et al. 2012; Chrysargyris et al. 2016), cytotoxic (Nikolić et al. 2014), antiseptic, anti-inflammatory, and analgesic properties (Chrysargyris et al. 2016), pharmaceutical (Biswas et al. 2009), ambient odors (Ciobanu et al. 2012), and tastes and fragrance (Ciobanu et al. 2012) are only a few of the biological characteristics of *Lavandula angustifolia*. Enzyme-assisted treatment can help release secondary metabolites from plant matrix. It offers the advantages of being environmentally friendly, simple to use, and efficient (Boukroufa et al. 2015; Hosni et al. 2013) (Dyk and Pletschke 2012). In this procedure, hydrolytic enzymes are used to break down cell membranes. The majority of hydrolytic enzymes are formed up of polymers that are highly complicated, such as cellulose, hemicellulose, lignin, and pectin (Boukroufa et al. 2015). Essential oils and plant extracts from the genus *Lavandula* have been used to cure a variety of diseases for millennia (Rai et al. 2020). The chemical composition of oils distilled from the two most commonly used species, *Lavandula angustifolia and Lavandula intermediate*, has been thoroughly studied (Rai et al. 2020). Although *L. angustifolia* oil and one of its main constituents, linalool, have been shown to have antibacterial activity against mites, grain weevil, aphid, and clothes moth (Hosni et al. 2013; Dyk and Pletschke 2012), most of the research is on oil’s antifungal activity. Essential oil volatiles have been used for therapeutic purposes for a long time. Because of the psychological and physiological benefits of inhaled volatile molecules, aromatherapy is thought to be therapeutic. Later implications are assumed to be mediated by the limbic system, notably the amygdala and hippocampus. Despite claims that inhaling lavender oil volatiles can improve a patient’s mood and sleep patterns, the actual therapeutic efficacy of lavender oil inhalation is still being contested (Kim and Cho 1999; Nelson 1997). This could be because many studies combine massage with lavender oil, making it impossible to tell whether the effects are attributable to massage or lavender oil absorption. Lavender (*Lavandula angustifolia*) has a pleasant, relaxing perfume and is high in phenolic components, especially gallic and rosmarinic acids. The mydriatic, antispasmodic, anticholinergic, analgesic, and relaxing effects of lavender have all been thoroughly researched. In the past, lavender was used to cure asthma, gastric ulcers, and Parkinson’s disease (Kim and Cho 1999).

## Historical or theoretical uses that lack sufficient evidence

Acne, alopecia, gas, hangovers, hypotension, analgesia, angio-protectant, anti-colic, anticonvulsant, antidepressant, antiflatulent, antifungal, anti-inflammatory (Nelson 1997), antimicrobial (Siurin 1997), antioxidant, appetite stimulant, asthma, balneotherapy (functional circulatory disorders), antipyretic, chronic bronchitis, cicatrizant, cordial, diabetes (Hardy et al. 1995), diuretic, douche, antiseptic, anxiety, cholagogue, choleretic, emmenagogue, exercise recovery, infertility, insect repellent, insomnia (Gabbrielli et al. 1988), lice, migraine, non-tubercular mycobacteria (NTM) (Adaszynska et al. 2011), parasitic infection, psychosis, rheumatism, toothache, varicose veins, Roemheld’s syndrome, rubefacient, vomiting.

## Chemical composition of lavender (Table 1)

**Table 1:** The chemical composition of essential oil as a percentage of the total oil composition. *nd*, not detected (Wells et al. 2018)

*Lavandula species (% range of total oil volume)*					
Constituents	*L. angustifolia*	*L. latifolia*	*L. stoechas*	*L. luisieri*	*L. x intermedia*
Linalool	6.97–44.4	27–61.1	0.08–2.7	0.2–3.1	21.1–32.3
Linalyl acetate	7.2–50.5	0.05–1.1	nd	nd	3.04–46.0
Borneol	0–6.2	0.16–5.9	0.8–1.4	nd	0–15.7
Alpha terpineol	0.6–3.5	0.1–2.95	0.09–0.66	nd	0–3.6
Lavandulyl acetate	2–3.5	nd	0–0.22	3.3–4.3	0–3.1
Oct-1-en-3-yl acetate	3.4	nd	nd	nd	nd
Cis-linalool oxide (furanoid)	3.2	0–0.6	0.05–0.1	0.7–1	nd
Cryptone	2.7	nd	nd	nd	Nd
Terpineo-4-ol	2.3–30.2	0.3–7.1	0.2–0.5	0	0–2.8
1,8-Cineole	0–6.03	6.6–34.9	8–52.7	2.4–43.2	5.2–26.1
Trans-linalool oxide (furanoid)	2.1	nd	0.01–0.05	nd	nd
Geranyl acetate	1.8	nd	0.01–0.02	0.2–0.4	nd
β-Myrcene	0.4–2.8	0.2–0.8	0.8	0-0.1	nd
β-Caryophyllene	1.5–4.3	0.5–1.9	0.2	nd	0–1.9
Octan-3-one	1.5	0.1–0.2	0.13–16.3	nd	nd
Gamma-cadinene	1.2	0.1–0.2	0–0.8	1.53	nd
ɑ-Cadinol	1.2	0–0.1	nd	0.5–2.4	nd
Cuminol	1	nd	nd	nd	Nd
Camphor	0–1.7	1.1–46.7	7.9–51.6	1.0–2.7	2.5–11.1
ɑ-Limonene	0.8–5.9	0.2–3.4	0.06–1.3	0.3–0.8	nd
Lavandulol	0–7.01	0.5–0.6	0	0.9–1.7	0–4.95
Neryl acetate	0.8	nd	nd	nd	nd
Camphene	0.4–0.6	0.2–5.3	0–4.4	0.1–0.2	0.1–1.2
*p*-Cymene	0.6	0–0.2	0.3	0.3–0.4	nd
Trans-pinocarveol	0.6	nd	0.03–0.1	nd	nd
Nerol	0.6	0.09–0.79	nd	nd	nd
Carotol	0.6	nd	nd	nd	nd
Hexyl acetate	0.4	nd	nd	nd	nd
β-Ocimene	0.4–21.2	0–1.3	nd	0–0.4	0–14.97
Cis-*p*-Menth-2-en-1-ol	0.4	nd	nd	nd	nd
Verbenone	0.4	0.08–0.7	0.2–1.5	1.7–2.1	nd
Cumin aldehyde	0.4	nd	0–0.01	nd	nd
β-Cyclocitral	0.3	nd	nd	nd	d
Daucol	0.3	nd	nd	nd	nd
ɑ-Pinene	0.3–0.5	0.6–1.9	0.2–2.1	2–3.4	0.2–0.6
Oct-1-en-3-ol	0.2	0–0.1	0–0.02	nd	nd
Cis-Verbenol	0.2	nd	nd	0–1.2	nd
Hexyl butanoate	0.2	nd	nd	nd	nd
Trans-carveol	0.2	0.0.1	nd	nd	nd
Gernial	0.2	nd	nd	nd	nd
(Z)-b-Farnesene	0.2	0–0.3	nd	nd	nd
ῳ-Cymene	0.1	nd	nd	nd	nd
Allo-ocimene	0.1	nd	nd	nd	nd
Myrenol	0.1	0.2–0.3	0–0.9	nd	nd
Geraniol	nd	0.06–0.8	nd	nd	nd
ɑ-Thajene	0.3	0–0.1	nd	nd	nd
β-Pinene	0.4	0.04–2.6	0.1–0.4	0.2–1.8	nd
Sabinene	0.5	0.02–1.2	0–0.7	0.1–0.2	nd
Gamma-4-carene	0.2	nd	nd	nd	nd
Para-mentha-1(7),8-diene	4	nd	0–0.46	nd	nd
Fenchone	nd	nd	2.9–68.2	2.9–6.6	nd

Lavender (*L. angustifolia*) contains essential oil, anthocyanins, ursolic acid, valeric acid, phytosterols, sugars, coumaric acid, glycolic acid, minerals, herniarin, coumarin, and tannins. Depending on the lavender varietal, different macronutrients are present (Colceru-Mihul et al. 2009). For the Munstead variety, potassium levels range from 17.7 to 23.9 g kg^−1^ dry matter (d.m.) for Lavender Lady. Climatic factors significantly affect the amount of calcium; the average value for lavender cultivated in Romania is 2.13 g Ca per 1 kg d.m. (Adnan et al. 2010). Pakistan has a value of 10.50 g Ca per kg d.m. (Góra et al. 2005). The calcium concentration of the Munstead variety is 13.8 g kg^−1^ d.m., compared to 8.10 g kg^−1^ d.m. for the Blue River variety. It has been discovered that the amount of trace elements varies according on the variety. The research in Góra et al. (2005); Adnan et al. 2010) also corroborated the low concentration of these elements, from 2.19 g to 4.25 g Mg per kg d.m. and 0.37 g Na per kg d.m. The range of zinc concentrations is 23.0 to 106.27 mg kg^−1^ d.m. (Góra et al. 2005; Adnan et al. 2010). The high amount of this micronutrient—25.7 mg kg^−1^ d.m.—was likewise confirmed by the investigation in Colceru-Mihul et al. (2009). 39.2 mg kg^−1^ d.m. was found for Lavender Lady Ellagance Purple.

Copper concentration ranges from 7.2 to 11.1 mg kg^−1^ d.m. and manganese concentration to 9.6 to 18.0 mg kg^−1^ d.m. for the Lavender Lady and Munstead subtypes, respectively. Ellagance Purple has the highest iron concentration (489 mg kg^−1^ d.m.), while Munstead has the lowest (137 mg kg^−1^ d.m.).

### Essential oil

The main compound of lavender is the essential oil, which is found in the oil glands between the sebaceous glands and the tiny hairs (*L. angustifolia*) on the surface of the calyx. Essential oils are offered in various concentrations ranging from 2 to 3%. It has a strong aroma of floral herbs and lavender, fruit, and wood aromas, and a bright yellow color. It is extracted by steam distillation or hydrodistillation (Prusinowska and Śmigielski 2014; Lawrence 2011). Genotype, growth site, climatic conditions, and reproductive and morphological characteristics all affect the qualitative and quantitative composition of lavender (*L. angustifolia*) essential oils (Smigielski et al. 2011). The most common chemical constituents of essential oils are linalool (9.3 to 68.8%) and linalyl acetate (1.2 to 59.4%). Essential oils extracted from fresh dried lavender flowers grown in Poland show a NIR profile most similar to that of French lavender (90.39% of fresh lavender and 97.65% of dried lavender) (Rajeswara Rao et al. 2006). The amount of linalool and linalyl acetate in the essential oil of lavender as well as their relative proportions determines its quality (higher than 1). Borneol, -terpineol, terpinene-4-ol, and lavandulol acetate, as well as caryophyllene and linalool oxides, are among the most common constituents. The majority of the compounds in this class are oxygenated monoterpenes (73.8%), with monoterpene alcohols making up 36% of them (Rajeswara Rao et al. 2006). Ocimene, cineol, camphor, and terpinen-4-ol adversely alter the distinctive herbal-rosy scent of lavandulol and lavandulol, which is characterized by a high concentration of lavandulol and lavandulol acetate (Smigielski et al. 2011). According to Lawrence (Smigielski et al. 2011), the scent of this essential oil is composed of acetic acid due to the presence of alcohol and its esters. The presence of (Z) Hex-3-enol and its esters raises the fresh green herbal floral tone.

Herbal and rustic scents are produced by Oct-1-en-3-ol and its esters. Fruity-fatty scents due to the presence of butyl and hexyl esters: linalool, lavandulol, and their esters (linalool acetate and lavandulol acetate) are responsible for the scent of fresh flowers. Monoterpene aldehydes and ketones produce herbal flavors. The sweet aromatic notes are due to santalene derivatives and sesquiterpenes. The change in scent is affected by the presence of pyridine. It is common practice to add cheaper alternatives to lavender essential oils. Synthetic chemicals or *Lavandula latifolia* essential oils, or hybrids of *Lavandula angustifolia* and *Lavandula latifolia*, are selected due to their high cost. Hydrolates are produced as a by-product of steam distillation of essential oils (Lawrence 2011; Śmigielski et al. 2013). Hydrolate is formed from plant water and process water and is also known as hydrosol or herbal water. Depending on the final volume, hydrolate has a strong herbal aroma, a sweet flower-herbal lavender scent, or an almost imperceptible lavender scent. The total number of volatile organic compounds in the hydrosol varies between 24.83 and 97.23 mg/100 mL hydrosols (Simsek et al. 2021). Linalool (39%) is the main ingredient, followed by terpineol (15%) and coumarin (7%). Hydrolate does not contain linalyl acetate, a chemical component found in large quantities (1.2–59.4%) in lavender essential oils. According to Migielski et al. (Rajeswara Rao et al. 2006), lavender (*L. angustifolia*) loses more than 40% of its essential oils after drying. When fresh lavenders are dried in a fluidized bed of a closed-circuit system equipped with a desiccant and heat exchanger at specific humidity, they produce more volatile and biologically active molecules and condensates from the plant. This is a process known as Fluidolat (Lawrence 2011). This physiologically active and unique compound is completely destroyed by traditional processing procedures. The aroma of lavender fluidolates is similar to that of lavender hydrolates, but it is stronger and more scented. VOCs (volatile organic compounds) in fluidolates range from 120.62 to 180.26 mg/100 mL fluidolate, depending on how dry the component is. The most prominent components are linalool (65.22–79.2%), terpinen-4-ol (6.3–16.4%), and lavandulol (0.8–4.4%). Monoterpene hydrocarbon oxygen derivatives account for the majority of the substances (80–91%), including monoterpene alcohols (47–61%). Linalyl acetate is also lacking in fluidolates.

## Biological activities

### Neurological effects of lavender

Lavender has also demonstrated efficiency in treating brain tumors. Silver nanoparticles and lavender extract combined (La-AgNPs) were studied in Caputo et al. (2016) for their effects on human brain tumor cells’ ability to proliferate and their ability to induce apoptosis (U87MG). They came to the conclusion that La-AgNPs in tumor cells can promote an increase in the expression of apoptotic proteins such P53 and caspases 3, 8, and 9. The study’s findings demonstrate that via altering the P53 apoptotic pathway, La-AgNPs can cause apoptosis in these tumor cells. La-AgNPs were recommended as a potential adjunctive therapy for glioblastoma in the study (Caputo et al. 2016). Similar research was conducted on two human glioblastoma cell lines by Chan et al. on the apoptotic, cytotoxic, and growth-inhibiting effects of low-dose lavender extract (U-87 MG and U-138 MG). According to the study, lavender oil in its diluted form can cause cancer cells to necrose and undergo apoptosis.

In a study conducted in PC12 cell culture, Caputo et al. (2021) compared the neurotoxic effects of A1-42 protein components—which play a critical role in the formation of Alzheimer’s disease—to the neuroprotective effects of lavender essential oil and its main active ingredient linalool. Their research shows that the proapoptotic enzyme caspase-3 synthesis and activity might be inhibited, along with morphological deviations and reactive oxygen levels in cells. By counteracting the effects of A1-42 protein components’ dysregulation of Ca 2 + homeostasis via voltage-gated calcium channels (VGCC) and NMDA receptors, lavender essential oil can reduce A1-42 protein components’ neurotoxicity (Caputo et al. 2021). As a result, it stops the dysregulation from causing reactive oxygen to be produced and activating caspase-3, which sets off an apoptotic cascade. They proposed linalool, the primary component of lavender essential oil, as a potential treatment for Alzheimer’s disease (Caputo et al. 2021). In a different investigation, Simsek et al. (Caputo et al. 2016) examined the in vitro antioxidant, anti-aggregative, and anti-acetylcholinesterase properties of lavender. They said that because lavender has antioxidant properties and is not cytotoxic, it can be utilized to treat neurodegenerative illnesses like AD. This herbal remedy opposes the fibrillation of A and hinders the development of plaque; however, it had no preventative effect on the activity of acetylcholinesterase. The neurotoxicity of glutamate is also decreased by the lavender extract, as well as the extracellular repositioning of *N*-methyl-d-aspartic acid receptors (Dobson and Giovannoni 2019).

The brain and spinal cord are affected by the debilitating condition known as multiple sclerosis (MS). In MS, the immune system targets the covering that protects nerve fibers (Fard et al. 2017). The effects of *Lavandula* essential oil on the gene expressions of brain-derived neurotrophic factor and interleukin-23 in peripheral blood mononuclear cells of patients with relapse-remitting multiple sclerosis were assessed in Seddighi-Khavidak et al. (2020). This study showed that *Lavandula* essential oil could protect against neuronal degeneration by increasing the gene expression of brain-derived neurotrophic factors in peripheral blood mononuclear cells of MS patients who are experiencing relapses and remissions (Seddighi-Khavidak et al. 2020). Seddighi-Khavidak et al. (Mehravar 2020) assessed the effect of lavender oil as an olfactory incentive and vestibular rehabilitation on the balance and function of daily life in MS patients. In comparison to the control group, the lavender treatment group performed better on the 29-item MS impact scale, Berg balance scale, Timed Up and Go TEST, and fall effectiveness scale—international tests. According to the study, utilizing lavender oil as an olfactory motivation while performing vestibular rehabilitation exercises reduces fear of falling and improves balance in MS patients by stimulating the insular brain (Mehravar 2020). The Wilcoxon test in this randomized, double-blind clinical research revealed a considerable difference in the tremor of the group receiving lavender treatment. In summary, this herbal medication can be taken alone or as a supplemental medication, and even the smallest dose of 80 mg is effective in reducing tremor in MS patients (Qneibi et al. 2019). The AMPAR (amino-3-hydroxy-5-methyl-4-isoxazole propionic acid receptor) is overactivated in several neurologic conditions as stroke, epilepsy, ALS, Alzheimer’s disease, and others. In a 2019 study, Qneibi et al. (Walsh and Wilson 2013) examined the effects of lavender essential oil on AMPAR activity and demonstrated that it changes this activity. In these illnesses, synaptic dysfunction and Ca2 + dysregulation are prevented by lavender because it functions as an AMPA receptor antagonist and inhibits glutamate from binding to it. They came to the conclusion that lavender essential oil might be employed as an AMPAR antagonist and a neuroprotective medication for the conditions known as neurologic illnesses (Walsh and Wilson 2013).

### Aromatherapy treatment

Each patient was given a 24-h rest interval of between treatment and a series of physical, behavioral, and psychological assessments. Physiological markers of stress were blood pressure, heart rate and rhythm, and respiratory rate, as well as behavioral response (motor activity, facial expressions, unconscious patient’s Glasgow Coma Score, etc.), of a subject during massage, aromatherapy, or rest. There was no significant difference. The aromatherapy group saw a significant reduction in anxiety during the first treatment session. There was no significant improvement in the second or third session, probably because other treatments were more effective than without aromatherapy. In a pilot study in Alaoui-Ismaili et al. (1997), long-term inpatients in neurology showed improved mood levels and reduced psychological symptoms after aromatherapy (tea tree, rosemary, and *L. angustifolia* oils).

These studies suggest that lavender aromatherapy helps ICU patients have a better experience without physical or behavioral side effects. Some authors have found a link between the scent of lavender and positive feelings. Lavender oil has a pleasant aroma that has been linked to alterations in the autonomic nervous system, according to Tysoe (2000). Tysoe (Millot and Brand 2001) investigated the effects of oil burners and lavender oil on those who worked or visited the ICU and discovered that 88% of those respondents thought lavender oil had a favorable impact on the ward. This shows that vapors of lavender oil can assist to mask the unpleasant scents that can occur in hospitals. Millot and Brand (Diego et al. 1998) discovered that the aroma of lavender increased the pitch of male and female voices, with female voices having the highest pitch. Pitch changes are associated with socially positive emotions, so voice changes can be used to measure mood (for example, happiness or joy). According to Masago et al. (2000), participants who inhaled 10% lavender oil for 3 min were much more relaxed and less anxious and have improved mood, and EEG showed higher alpha intensity. Masago et al. (Degel and Köster 1999) also found that inhalation of lavender produces an EEG pattern that indicates how relaxed the patient is. Diego et al. (Masago et al. 2000) also observed that the use of lavender oil aromatherapy improves the speed and accuracy of mathematical calculations. This may explain why the lavender skull cap was previously thought to increase intelligence. The study in Kilstoff and Chenoweth (1998) confirms the accuracy of these conclusions. Subjects underwent a series of tests in a room with the scent of essential oils. They were unaware that the room was scented, but the room in the lavender-scented room was better than the room in the jasmine-scented or unscented room. According to one study, patients who received a 10–15-min hand massage blended with three oils containing lavender at a dementia care center in New South Wales, Australia, significantly improved in all areas studied. Benefits include happiness, increased arousal, reduced aggression and anxiety, and improved sleep patterns (Lindsay et al. 1997). According to a crossover study in Buckle (1999), hand massage with lavender oil did not appear to raise the attention of individuals with severe learning disabilities. Based on these findings, lavender oil can be used alone or in combination with other oils to treat a variety of illnesses, and is safe, effective, and easy for both conscious and unconscious patients. In addition, rooms with a scent of lavender oil are likely to be considered a comfortable environment, which can be very useful in some medical scenarios. The only caveat to this statement is the lack of information on the type and amount of lavender oil used due to the different oils and treatments used, and the indistinguishable massage effect from those caused solely by odor, and that the sample size is small. These results can be difficult to interpret. Still, lavender oil seems to have the potential to benefit patients, visitors, and staff in a variety of medical environments, especially when combined with massage.

Aromatherapy is recommended for chronic or refractory pain (Brownfield 2013). Lavender oils high in 1,8-cineole, such as Latifolia, have been shown to be effective analgesics. Buckle (Brownfield 2013) cited a number of research that demonstrate aromatherapy, with or without massage, can lower pain perception and the need for standard analgesics in both adults and children. Almost all investigations, including this one, were insufficient, uncontrolled, or lacked the customary scientific rigor associated with clinical trials. According to a quasi-experimental crossover study, lavender oil (*L. angustifolia*) reduced pain perception in patients suffering from rheumatoid arthritis and improved sleep quality and well-being (Ghelardini et al. 1999). The results of the study are based on the patient’s subjective assessment of pain, sleep, and well-being, and are not based on quantitative data from visual analog scales that did not show pain relief or sleep improvement. In vitro and in vivo studies of *L. angustifolia* oil and linalyl acetate and linalool have been shown to have a local anesthetic effect (Cavanagh and Wilkinson 2002b). According to these authors, the mechanism of action is associated with anti-muscarinic activity and/or blockade of ion channels (Na^‡^or Ca2^‡^). Because aromatherapy is a popular treatment among caregivers (Kite et al. 1998), palliative care facilities are more likely to use complementary therapies to improve the patient’s mood. Like other essential oils, lavender can help with cancer and chemotherapy side effects such as pain, hair loss, and anxiety. Aromatherapy has also been shown to help patients in palliative care (Huang et al. 2012). On the other hand, many of these studies lacked accuracy in describing the oils used, confusing comparisons between works by different groups, and many may be using different lavender oils. All future clinical investigations should include the exact origin of the oil utilized in the study, as well as the fluid’s GC/MS profile, or, if available, the percentage composition of the major components.

#### Mechanism of action of lavender in the nervous system

Several researches were done to determine the mechanism of lavender’s activity in neural tissues. Lavender’s activity in brain tissue has been explored in a variety of ways. Lavender inhibited the inflammatory response caused by lipopolysaccharide in human monocyte THP1 cells, which may be linked to HSP70 expression (Wang et al. 2012). In lavender (Salah and Jäger 2005; Perry et al. 2000) and linalool (Perry et al. 2003; Savelev et al. 2003), both antioxidant and cholinergic effects have been observed. At the neuromuscular junction, linalool reduced the release of acetylcholine and altered ion channel activity (Wang et al. 2012). These data suggest that lavender has anticholinergic, neuroprotective, and antioxidant properties that may help treat Alzheimer’s disease. The neuroprotective effect of lavender oil on cerebral ischemia/reperfusion injury is believed to be due to its antioxidant properties (Re et al. 2000). Intraperitoneal injection of lavender into the olfactory bulb of rats enhanced rotarod activity and increased D3 subtype dopamine receptors (Sousa et al. 2010). Lavender oil has also been shown to increase inhibitory tone of the nervous system and alter GABAergic neurotransmission, especially at GABAA receptors (Kashani et al. 2011; Umezu 2000). The cholinergic system can cause lavender’s analgesic, anxiolytic, antidepressant, and anticonvulsant effects (Yamada et al. 1994; Shaw et al. 2007). Fos is a nuclear transcription factor protein that is encoded by the c-fos immediate early gene and is an early marker to neuronal activation. It acts as a transcription factor that regulates the expression of genes associated with efficient adaptation to a particular environment. Lavender oil reduced c-fos expression in the paraventricular and dorsomedial hypothalamic nuclei of the hypothalamus (Aoshima and Hamamoto 1999). Lavender oil dose-dependently suppressed histamine release and anti-DNPIgE-induced tumor necrosis factor alpha production in mouse peritoneal mast cells (Shen et al. 2005). Lavender oil stimulates the parasympathetic nerves, which suppress the sympathetic nerves that innervate the white and brown adipose tissue, as well as the adrenal glands (Tanida et al. 2007) (Hammer et al. 1999). Through histaminergic responses, the aroma of lavender oil, particularly its ingredient linalool, stimulates the autonomic nerves of rats, lowers lipolysis and heat production (energy expenditure), and increases hunger and weight (Hammer et al. 1999). By activating H3 receptors, lavender may reduce sympathetic nervous system activity and lipolysis. The lipolytic response to lavender oil and the tyrosine phosphorylation of BIT are mediated by the hypothalamic suprachiasmatic nucleus and histamine neurons (a brain immunoglobulin-like molecule with a tyrosine-based activation motif that is a member of the regulatory signaling protein family) are implicated in the relevant signal transduction (Hammer et al. 1999).

### Antimicrobial activity

Many bacteria and fungi had been observed to be effective against lavender oil (basically *L. angustifolia*) (Antonov et al. 1997). Antibiotic-resistant bacterial infections have additionally been connected to crucial oils like lavender. *L. angustifolia* oil was found to be of in vitro interest with a attention rate of less than 1%, in contrast to MRSA (methicillin-resistant *Staphylococcus aureus*) and VRE (vancomycin-resistant *Enterococcus faecalis*) (Siurin 1997). Antifungal agents have been found in both oils and vapors. In the fungus *Botrytis cinerea*, *L. angustifolia* (1% and 10%) suppressed conidium germination and germination tube growth at doses up to 1000 mg/mL L (Daferera et al. 2000), although *L. angustifolia* did not affect the conidial production of *Penicillium digitatum* (Inouye et al. 1998). The oil of *L. angustifolia* changed into located to be extra green than hyphal proliferation at inhibiting germ tube growth. Surprisingly, one research found that gaseous contact with lavender oil (*L. angustifolia*) inhibited the growth of four filamentous fungi but not solution contact (Inouye et al. 2000). The direct binding of gaseous oil to the aerial mycelia that made up the spore-forming organ was claimed to be the reason. Solution touch was said to induce in reality little binding. When the activity of two primary ingredients was studied, linalyl acetate was shown to be capable of reducing spore formation, while linalool was found to be effective for inhibiting germination and fungal growth. The respiratory suppression of aerial mycelia seems to be the cause of spore formation inhibition. *Aspergillus fumigatus* mycelial growth has also been shown to be inhibited by lavender vapor, albeit the impact was short-lived and the dose needed (63 mg/mL air) was higher than that of tea tree oil, lemon grass, cinnamon bark, and thyme oils (Haag and Gould 1994). The direct deposition of essential oil on fungal mycelia, as well as an indirect action via absorption through the agar medium, was attributed to the suppression of apical growth by vapor contact. According to these investigators, vapor treatment has an advantage over solution treatment in that a lesser amount of essential oil can limit microbial growth while simultaneously functioning as an effective sporulation inhibitor. The vapor concentration and duration time are critical factors in the prevention of pathogen growth by gaseous treatment with essential oils. In this regard, it was observed that gaseous contact activity was primarily determined by the maximum vapor concentration early in the incubation process, and that maintaining a high vapor concentration for lengthy periods of time was not required. Linalool had an effective vapor concentration of 0.7 mg/mL of air against *T. mentagrophytes*, which was higher than that employed in aromatherapy (Lis-Balchin et al. 1998). Lavender oil has been a subject study around the world to identify and isolate its chemical components. This study will help identify the oil’s biologically active constituents and determine whether the “mixed” components have any synergistic benefits. While it is well-known that the main constituents of lavender oil play a significant role in its biological activity, it has also been revealed that the antimicrobial activity of different types of lavender oil is not all related to these major constituents, and little is known about any synergistic relationships between the oil constituents. Studies looking into the link between lavender’s biological activity and its chemical composition, for example, showed no link between linalool or linalyl acetate content and antibacterial or antifungal activity (Pattnaik et al. 1997). Indeed, there was a significant variation between different lavender samples; for example, lavender from Bulgaria (51.9% linalool, 9.5% linalyl acetate) was effective against 23 of 25 bacteria, whereas lavender from France (29.1% linalool, 43.2% linalyl acetate) was only effective against 13 bacteria. In addition, the two oils demonstrated differing effects on the fungi *Aspergillus niger* and *Fusarium culmorum*. Two Bulgarian lavender samples extracted using different methods (steam distillation vs supercritical carbon dioxide) demonstrated similar antibacterial activity but varied antifungal activity, implying that different oil components may be responsible for the activity against various pathogens. Pattnaik et al. (Inouye et al. 2001) discovered that linalool, a key component of lavender oil, inhibited 17 of the 18 bacteria (both Gram-negative and Gram-positive) and 10 of the 12 fungi (filamentous and non-filamentous) examined. Data on antibiotic activity in vitro, on the other hand, is frequently difficult to compare. When looking at the literature on the antimicrobial action of essential oils, such as lavender, a variety of approaches have been used. The most commonly used procedures are disk/well diffusion or agar/broth dilution method. Antibiotic activity data in vitro, on the opposite hand, is normally hard to compare. The antimicrobial activity of essential oils like lavender has been studied in the literature. The disk/nicely diffusion approach and the agar/broth dilution approach are the two commonly used methods. The use of diffusion and dilution methods had been proved to be correct, and they are now generally utilized in antibacterial susceptibility testing. However, it is really well worth noting that the compounds tested by these techniques are usually hydrophilic; therefore, the tests have been tailored to this condition. Because essential oils are volatile, insoluble in water, viscous, and complicated, the simple assays mentioned above are insufficient for antimicrobial testing. Inoculum size, medium utilized, usage of sealants, and surfactants and solvents like as Tween, dimethyl sulphoxide, and ethanol are among the variables that affect technique. The minimal inhibitory concentrations (MIC) of each *L. angustifolia* oil and linalool in opposition to fungus were reduced by more than four times when the medium was closed to keep the critical oil contents from evaporating during incubation (Lis-Balchin et al. 1998). As a result, several approaches of assessing crucial oils are actually getting used without standardization, necessitating the improvement of a consistent, robust, and repeatable procedure. Due to those issues, direct evaluation of published records is nearly impossible. The lack of detailed data on which lavender oils had been applied and which particular kind the oil derived from makes matters even harder. According to current findings obtained, specific lavender species have an extensive variety of antimicrobial activities, with a few having excessive antibacterial/antifungal activities and others having none at all. Antibiotic resistance was studied in Gram-positive, Gram-bad, spore-forming, and Gram-bad microorganism, yeast, and fungus. The antibacterial activities of lavender oil seem to be affected by a number of factors, such as species, cultivation factors, and distillation methods (Kite et al. 1998). Despite its antibacterial activities, a few people are skeptical of lavender oil’s scientific use. In vitro, lavender oil (*Lavandula angustifolia* and *Lavandula latifolia*) has antimicrobial interest akin to tea tree, with MIC values of 0.16% against *Haemophilus influenza*, 0.32%, against *Streptococcus pyogenes* and *Staphylococcus aureus*, and greater than 0.32% against *Escherichia coli* (Moon et al. 2006).

### Anti-parasitic activity

The efficacy of essential oils in the treatment of mammalian infections is currently unknown. Essential oils have historically been recommended for the treatment of mammalian parasitic infections. Anti-parasitic activities of two essential oils extracted from *Lavandula angustifolia* and *Lavandula intermediate* against the protozoan human flagellates *Giardia lamblia* and *Trichomonas vaginalis* and the fish infection *Hexamita* inflate have been studied (Upcroft and Upcroft 2001). Despite recent advances in antibacterial chemotherapy, treatment of medically, veterinary, and socioeconomically important parasitic infections remains difficult (Upcroft and Upcroft 1998). *Giardia* and *Trichomonas* are two common human infectious diseases found worldwide. *Giardia* causes symptoms such as chronic and acute diarrhea and failure to thrive in children, affecting an estimated 200 million people worldwide each year (Furness et al. 2000; Cudmore et al. 2004). *Giardia* is abundant not only in human and animal populations but also in the environment, so eradication is always difficult. Instead, giardiasis needs to be treated. Most parasite illnesses have limited treatment options, and resistance is on the rise (Upcroft and Upcroft 1998; Vesy and Peterson 1999; Patel et al. 2000). *Trichomonas vaginalis*, the most frequent non-viral infection, has been linked to infertility, early birth, low birth weight, high infant mortality, and, more recently, increased HIV susceptibility (Vesy and Peterson 1999; Schwebke and Burgess 2004; Biagini et al. 1998). It accounts for 11% of all cases of non-gonococcal urethritis in men and causes prostatitis, epididymitis, and infertility (Vesy and Peterson 1999). *Hexamita* is a heterotrophic parasitic flagellate that infects a variety of animals, including fish, crabs, and birds (Perrucci 1995). It does not pose a significant health risk to humans, but it does have a significant impact on the marketability of farmed or commercially farmed fish. *Hexamita* infection results in a marked loss of vitality and appetite, as well as a darker color and bloating, usually caused by the involvement and swelling of the gall bladder (Upcroft and Upcroft 2001).

### Pesticidal activities of lavender oil

Studies have demonstrated that both linalool and *L. angustifolia* oil have ascaricidal properties. Both direct contact with mites and inhalation of volatiles were linked to ascaricidal effects in a study of the effects of *L. angustifolia* oil and linalool on *Psoroptes cuniculi* (Plarre et al. 1997). Lavender oil or powdered leaves and flowers are effective against mites, grain weavers, aphids, and clothes moths and can be used as commercial and household pesticides (e.g., in grain elevators) (Hori 1998; Sugiura et al. 2000). Lavender oil has also been tested as a treatment for mites that cause psoroptic mange in sheep with promising results (O’Brien 1999).

### Dermatological uses

Despite the fact that lavender oil was considered particularly beneficial during World War I, there is little evidence that it helps to accelerate wound healing and reduces scarring. Other skin conditions associated with lavender oil include psoriasis, dermatitis, and eczema. Topically applied lavender oil has also been shown to block some of the allergic pathways (Nelson 1997). As with other essential oils, the use of lavender raises concerns about possible allergic reactions and skin irritation. Cavanagh and Wilkinson (Cavanagh and Wilkinson 2002a) claim that *L. latifolia* and *L. angustifolia* do not cause skin sensitization and are relatively moderately irritating to the skin, and that *L. angustifolia* does not cause phototoxicity in the well-cited essential oil safety studies. On the other hand, these authors do not provide evidence to support their claim. In Japan, up to 13.9% of patients developed contact dermatitis after 9 years of exposure to lavender oil (Coulson and Ali Khan 1999). In addition, later in their study, these researchers noticed an increased incidence of contact dermatitis associated with higher use of dry lavender products. This period (1997) correlates with the global increase in the use of essential oils, which may increase product usage, thereby causing the frequency of contact dermatitis and other skin problems. There have also been cases of contact dermatitis from using lavender pillows (Benito et al. 1996) and cross-sensitivity responses with other Labiatae family members (Anderson et al. 2000). Eczema in children manifests as skin irritation that is difficult to treat and causes significant distress to both the kid and the parents. Lavender and other essential oils have been tried as a replacement for traditional AL medications (e.g., topical steroids), which have been proved to be inefficient. For the treatment of eczema with massage and bath water, the efficacy of a series of essential oils containing lavender (6 drops of 3 oils in a 1:1:1 ratio) has been investigated in Hutchins et al. (1985). After 3 weeks of treatment, despite the limited pilot size (16 children), both massage with essential oil and massage only showed a significant reduction in night time discomfort and irritation. Aromatherapy massage and massage only groups were not statistically different. However, since the authors point out, it is difficult to distinguish the tactile effect of the massage from those of the aroma. According to Kite et al. (1998), 14 out of 15 people with moderate to severe psoriasis who were given essential oils containing lavender improved their physical symptoms, self-confidence, and self-esteem. The family also benefited from treatments that either inhaled vapors from the oil or helped apply them. Note that this seems to be an impressive result, and not a clinical study report, and does not provide an objective assessment of the mechanism of treatment or severity before and after treatment. Lavenders such as rosemary oil, cedarwood, and thyme are said to help people with alopecia restore their hair. Hay et al. (Hay et al. 1998) used a randomized, double-blind, controlled experiment to investigate this effect. Eighty-six alopecia areata patients rubbed a mixture of *Rosmarinus officinalis* (114 mg), *Cedrus atlantica* (94 mg), *Thyme vulgaris* (108 mg), and *L. angustifolia* (108 mg) oil onto their scalps each night, and hair growth was measured at 3- and 7-month intervals.

### Relieving perineal discomfort following childbirth

Women are exposed to a lot of stress. In hospitals, perineal ice packs, bathing, and administration of drugs such as paracetamol and co-dydramol (Sleep and Grant 1988) are common. Midwives have traditionally used salt or Savlon® as the water component of the bath to prevent infections, improve healing, and thereby relieve discomfort, but have proven ineffective (BabashahiKohanestani et al. 2013). Description of the properties that reduce perineal discomfort in lavender oil has been shown to be effective; it is safe, comfortable, and non-invasive to reduce perineal discomfort in the mother after childbirth. It can be a targeted and relatively inexpensive method. Midwives can recommend it, and mothers can give it themselves (Cavanagh and Wilkinson 2002a).

### Hypnotic/sleep and vital signs

The impact of lavender aromatherapy on sleep quality was examined in four research. A RCT study found that colorectal cancer patients’ quality of sleep before surgery was enhanced by 10 min of massage using the “Back Massage Guide” and 5% lavender oil (Hui et al. 2010). Another study with an RCT design demonstrated that using three drops of 100% lavender essential oil for 7 days before bedtime for 20 min, inhalation aromatherapy enhanced the quality of sleep in cancer patients admitted to the oncology unit (Buchbauer et al. 1991). Additionally, cancer patients’ sleep quality was enhanced by inhaling aromatherapy with lavender essential oil for 5 min each night for a month following chemotherapy (Hussain et al. 2011). According to the 45 study by Soden et al., giving patients with cancer in palliative care units a 30-min back massage using 1% lavender essential oil every week for 4 weeks enhanced their sleep scores.

The impact of lavender aromatherapy on the various vital sign indicators was assessed in three trials. In the study by Goepfert et al. (Kritsidima et al. 2010a), breathing and heart rate as well as diastolic, systolic, and mean arterial pressure all dropped after palliative cancer patients inhaled aromatherapy using three to four drops of lavender oil for 10 min. Additionally, it was discovered that inhaling aromatherapy for 60 min while using 30 drops of 3% lavender essential oil reduced the heart rate and blood pressure in cancer patients receiving in-home hospice care (Skoglund and Jorkjend 1991). One study, however, found that inhaling 1% lavender oil or 1% linalyl acetate with 1 mL had no effect on blood pressure and heart rate in colorectal cancer patients undergoing surgery (Ghelardini et al. 1999).

### Pain

The effects of lavender aromatherapy on pain severity were examined in four research. Inhaling lavender essential oil at a concentration of two drops for three nights during chemotherapy for AML patients reduced their level of pain, according to a quasi-experimental study (Yayla and Ozdemir 2019). Additionally, the research by Yayla and Ozdemir (Yu et al. 2017) shows that the pain caused by putting a needle into an implantable intravenous port catheter could be lessened by inhaling aromatherapy using three drops of lavender essential oil for 3 min. Additionally, it was demonstrated that inhaling 1 mL of 1% lavender oil or 1% linalyl acetate for roughly 20 min after surgery reduced the severity of the postoperative discomfort (Soden et al. 2004). However, one study showed that a 30-min back massage with 1% lavender essential oil given to cancer patients in palliative care units once a week for 4 weeks had no effect on their level of pain (Rees et al. 1979).

### Local anesthetic activity

The chemical components responsible for this biological activity are essential oils, some of which are used to treat gastrointestinal spasms (Mazzanti et al. 1998*).* In vitro studies of the antispasmodic effects of essential oils from the above plants have shown that they can suppress seizures induced by various seizure-inducing substances through pharmacological mechanisms that suggest non-specific antagonistic interactions (Broucke and Lemli 1980). Essential oils are thought to slow neurotransmission by interacting with the plasma membrane lipid bilayer, limiting Ca^++^ influx or preventing increased Na^+^ permeability (Karan 2019). In addition, menthol is used in analgesics and local anesthetics, and eugenol is a membrane stabilizer with local anesthetic effects (Cavanagh and Wilkinson 2002b).

The investigated compounds were obtained from *L. angustifolia* Mill. (Lamiaceae), a medicinal plant traditionally used as an antispasmodic, sedative, and antiseptic drug (Hori 1998), and from *Citrus reticulate* Blanco and *Citrus limon* (L.) Burm. f. (Rutaceae) that have no medical uses. A previous study showed that linalool has a noteworthy antimicrobial activity in vitro while limonene is poorly active (Cavanagh and Wilkinson 2002b).

### Antibiotic and spasmolytic activity

Lavender oil appears to have antibacterial effects based on preliminary results from in vitro studies. However, the results are not considered therapeutically relevant as no animal or human studies have been tested. Gabbrielli et al. demonstrated in vitro activity of lavender oil (*L. angustifolia* and *L. latifolia*) against various strains of non-tubercular *Mycobacterium* (Adaszynska et al. 2011). Nelson et al. found that lavender oil was effective against methicillin-resistant *Staphylococcus aureus* (MRSA) and vancomycin-resistant *enterococci* (VRE) in the range of 2 to 0.12% (v/v) (Siurin 1997). Preliminary evidence from animal and in vitro experiments suggests that inhalation of lavender oil may have a convulsive effect. On the other hand, human evidence is limited. In animal studies, lavender species have been observed to reduce muscular spasms in the ileum and conjunctiva (Lis-Balchin and Hart 1999, 1997).

### Anxiety

Lavender oil has been researched for usage in the extraction of third molars and is a beneficial adjuvant, according to Karan et al. and Nardarajah et al. (2018; Kritsidima et al. 2010b). It may be difficult for the patient to deal with dental anxiety while they are waiting to receive treatment. Lavender essential oil has been reported to lessen general anxiety throughout this waiting period (Saeki 2000).

Evidence of lavender aromatherapy as an anxiolytic is generally limited. Some methodologically flawed studies have shown no effect and have produced a variety of results. However, the volume of the data suggests that there is a slight positive benefit in reducing anxiety. This case is enhanced by doing more research in the form of well-designed randomized trials. However, there are many challenges in designing a blind or placebo control for the study of sensory treatment. There are several challenges that need to be addressed before achieving compelling results. Saeki et al. attempted to demonstrate that lavender aromatherapy via footbath produced anxiolytic effects compared to placebo (Motomura et al. 1999). A pre- and post-study of 10 subjects found that a warm footbath with lavender oil was associated with small but significant changes in autonomic activity. However, the results are difficult to interpret due to insufficient disclosure of methods and analyses. Dunn et al. conducted a randomized single-blind study on 122 ICU patients randomly assigned to one of three groups: grape seed oil body massage, lavender oil body massage, or undisturbed rest (Dunn et al. 1995). Physiological endpoints included blood pressure, heart rate, and respiration, and psychological endpoints were assessed using any 4-point scale. One to three 30-min sessions were scheduled at 24-h intervals. Each patient was given at least one session and 66 patients were given three sessions. Patients who received lavender oil massage reported much less anxiety than those who slept after the first session. This distinction was not maintained in subsequent sessions. It is unknown to what extent the results were affected by the lack of double blindness or the high dropout rate. Motomura et al. conducted a study on 42 other students who were divided into three groups. One is a “stressful state,” the other is a “stressful state” with the scent of lavender, and the third is a “stress-free state.” The Japanese version of the Cox and McKay Stress/Awakening Adjective Checklist was used to assess stress (Itai et al. 2000). The results in the lavender group were significantly lower than those in the stressed control group. On the other hand, blinding and randomization have not been fully studied. Itai et al. evaluated the effect of lavender oil on mood using the Hamilton Rating Scale for Depression (HAMD) and the Hamilton Rating Scale for Anxiety (HAMA) (Buckle 1993). Compared to natural smell (baseline), lavender was observed to decrease anxiety as evidenced by the HAMA scale (*P* = 0.05). Lavender did not affect the patient’s HAMD assessment compared to baseline. There was no noticeable difference in the HAMA and HAMD ratings between the lavender and unscented settings. Buckle (Seeman 1980) studied the therapeutic effects of lavender oil in 28 inpatients of two different types (*L. angustifolia* and *L. burnatti*). A semi-structured interview was conducted a few days after treatment to collect qualitative and subjective data. The sedative effect of *L. burnatti* was shown to be significantly greater than in comparable studies.

### Role in apomorphine-induced ejaculation in male

Traditional medicine has used many plants as sexual arousal and ejaculatory regulators. *Lavandula angustifolia* Mill. is a well-known and widely used herb that has a variety of physiological effects, including relaxation, sedation (Cavanagh and Wilkinson 2002a), anti-conflict (Shaw et al. 2007), and altered sexual activity. Linalool, an important component of lavender extract, has been shown to act on glutamatergic and GABAergic receptors (Elisabetsky et al. 1995), as well as dopaminergic receptors. Based on the psychological and physiological abilities of this plant, as well as the various systems involved in ejaculatory behavior, the metabolites of *L. angustifolia* may be beneficial for ejaculation. D1 and D2 (dopamine) receptors have been shown to have a dopaminergic effect by apomorphine (Ahlenius and Larsson 1990). The dose of apomorphine used in this study (0.4 mg/kg) was previously reported to elicit a significant ejaculatory response (Abuhamdah and Chazot 2008). In this study, pretreatment with *L. angustifolia* extract (100 mg/kg) significantly reduced ejaculation with apomorphine. Linalool, the main component of *L. angustifolia* extract, has anticonvulsant activity and modifies NMDA receptors in glutamate-related seizure models (Brum et al. 2001; Moura Linck et al. 2009). At the neuromuscular junction of mice, potassium-stimulated glutamate release was also blocked, altering the dynamics of nicotinic receptor ion channels (Rajeswara Rao et al. 2006). On the other hand, *L. angustifolia* is associated with anxiolytic effects (Jannini et al. 2005) and its role in sexual behavior has been studied (Okuyama et al. 2004). Linalool affects not only dopamine release from striatal slices of the rat brain (Yamada et al. 2005), but also plasma dopamine levels in ovariectomized female rats (Adams et al. 2007). A double cholinergic effect was also found in *L. angustifolia* extract (Zarrindast et al. 1994). Dopaminergic and cholinergic neurotransmitters are associated with ejaculatory habits (Zaringhalam et al. 2010). According to Issa et al. (2011), the physiological effects of *L. angustifolia* on ejaculation are mediated, at least in part, by disruption of dopaminergic and cholinergic neurotransmission.

### Management of diabetic dyslipidemia

*Lavandula angustifolia* is widely cultivated in Northern Jordan and is used for a variety of traditional medicinal and cosmetic purposes. Lavender has long been recognized for its therapeutic potential in the treatment of type 2 diabetes and is currently being tested. Lavender extract has long been used in the treatment of anti-diabetes in Northern Jordan. Lavender (*Lavandula angustifolia*) native to Jordan has long been used in Jordanian traditional medicine to treat diabetes. Issa et al. (Lis-Balchin and Hart 1999) investigated the usefulness of plant methanol extracts in the treatment of diabetic dyslipidemia. According to the result, *L. angustifolia* reduced the activity of both hormone-sensitive lipase (HSL) and pancreatic lipase (PL) in vitro. The results showed that the lavender extract dose-dependently suppressed HSL activity at 175.5 g/mL IC50. In addition, 56.5 g/mL IC50 suppressed PL activity in a dose-dependent manner. Such inhibitory effects may be associated with the presence of rosmarinic acid (125.2 and 51.5 g/mL IC50s for PL and HSL, respectively) and gallic acid (10.1 and 14.5 g/mL IC50s for PL and HSL, respectively).

Interestingly, the inhibitory pattern of the lavender on the enzymatic activities of HSL and PL matched the inhibitory pattern of orlistat (Lis-Balchin and Hart 1999).

### Effects on other body systems

Lavender has an antispasmodic action on the intestine and uterine smooth muscle as well as the influence on the central nervous system by animal studies. These mild muscle relaxation activities may contribute to human relaxation effects (Cornwell and Dale 1995). Anti-spasmolytic effect of lavender was mediated as an increase in intracellular bearing (Cornwell and Dale 1995) instead of adrenergic or cholinergic receptor, nor actions at calcium or potassium channel activity. It is still unknown whether this is associated with receptor activation or the relaxation effect in humans (via olfactory and limbic system) or peripheral transmission results. These smooth muscle measurements, thus blood vessel dilation, can also explain the blood pressure–reducing effect of lavender that can be connected to the lever-oral component of lavender oil (Kite et al. 1998). When the lavender oil is added to the bathing water, it is used for midwives to relieve pain and discomfort after pregnancy. According to a large clinical study, mothers who used lavender oil 3–5 days after delivery had less difficulty (Dale and Cornwell 1994; Nagai et al. 2000). Lavender oil is also used in many delivery rooms for its relaxing effect. Traditional lavender poultices have been placed on the hips to relieve muscle tension during labor and on the abdomen to aid placental drainage. Inhaling oil has beneficial anxiolytic and relaxing effects for the mother, but the latter two uses have not been scientifically proven. Essential oil flavors (such as rosemary and lavender) are recommended as performance-enhancing aids in exercise training. Cavanagh and Wilkinson (Kite et al. 1998) studied oxygen consumption, heart rate, respiratory exchange rate, and subjective exercise in a group of five during submaximal activity (treadmill walking). Flavors were observed at 3-min intervals both before and during exercise. When lavender was compared to other essential oils (basil, rosemary, peppermint) or placebo, there was no significant difference in any of the metrics, indicating that the scent had no physical effect during submaximal activity. Other considerations, such as maximum sustained exercise and psychological benefits, that could indirectly improve performance were not included in this study.

In addition, although these participants believed they had exercised force (8–9 on a 1–10 scale), physiological data suggest they did not. This can lead to results if these findings apply to appropriate and well-trained athletes. A study of the physical response to exercise (rhythmic handgrip) (Romine et al. 1999) found that inhaling a pleasant scent (lavender, lemon, rose, or another oil of the subject’s choice) during exercise significantly increased diastolic blood pressure. No other parameters (e.g., finger pulse amplitude, systolic blood pressure, and respiratory rate) were affected. Unfortunately, no attempt was made to distinguish between the different oils used. According to another study, lavender aromatherapy did not affect cardiovascular markers after moderate motor recovery (blood pressure, heart rate, pulse pressure) (Jäger et al. 1992). According to the author, the lack of statistical significance is due to the size of the small group (10 participants), not to the missing effect. All authors point to the size of a small group which is a major obstacle in determining whether lavender or other essential oils are therapeutically effective during exercise or post-exercise recovery.

## Kinetics

### Topical

Lavender oil absorbs very fast into the skin. Five minutes after topical treatment, components linalyl acetate and linalyl glucoside are detectable in the blood, peaking at 19 min and step by step declining after 90 min (Ripple et al. 2000).

### Oral

Perillyl acid (PA) and dihydroperillyl acid (DHPA) are generated when the components limonene and perillyl alcohol (POH) are digested. High levels of PA are detectable at 12 and half hours in rats fed a meal regimen containing POH or limonene, peak ranges of DHPA are seen at 23 and half hours, and every metabolite has a half-life of 12 h (Haag and Gould 1994). POH, PA, and DHPA can all be found in the urine of people who consume large amounts of POH. Within the first 24 h, about 9% of the total dose may be recovered. The most frequent metabolite is PA, which accounts for around 1% of all POH collected. Nutritional use does not appear to affect POH consumption (Snow et al. 2004).

## Dosing

Adult dosage recommendations (18 years and older): Take 1–2 tablespoons of oral herbs orally as tea (primarily based totally on anecdotal and professional opinion). Soak 2 teaspoons (10 g) of leaves in 250 mL (1 cup) of boiling water for 15 min to make tea. Inhalation: Inhale two–four drops of essential oil vapor into 23 cups of boiling water. Aromatherapy may be used on an everyday basis or on a case-by-case basis (primarily based totally on anecdotes and professional opinion). (Topical/Infusion) Bath addictive: Bath additives containing 6 drops of lavender oil (without a selected brand) have been examined for perianal pain for the duration of childbirth (Nagai et al. 2000). If you need to take advantage of complete flower, add 1/4 to half cup of dried lavender flowers to the bathtub water (primarily based totally on anecdotes and professional opinion). Massage therapy: One–four tablespoons of base oil for massage therapy (primarily based totally on anecdotes and professional opinion).

### Human studies

Linalyl and linalyl acetate can cause central nervous system depression because they are easily absorbed through the skin after topical treatment with massage. Recent studies of lavender oil aromatherapy in patients with dementia show little evidence that olfactory aromatherapy reduced the excitement of people with severe dementia, requiring the application of essential oil epidermis for optimal results (Heuberger et al. 2004). Transdermal administration of linalool, one of the main components of lavender oil, reduced systolic blood pressure and skin temperature, but did not affect subjective health compared to the placebo-treated group (Gamez et al. 1990).

The improvement of sleep is another benefit of lavender oil application. Lavender aromatics have been shown to help babies and the elderly sleep better (Basch et al. 2004; Elisabetsky et al. 1995). Additionally, prolonged slow-wave sleep lasts longer when exposed to lavender scents while sleeping (Brandao 1986). Reducing anxiety is a therapeutic benefit that is directly tied to improved sleep, and lavender essential oil has been shown to have anxiolytic properties in numerous studies. Lis‐Balchin and Hart (Lis-Balchin and Hart 1999) connected the sedative and calming effects of lavender oils with their effect on the central nervous system delivered via the olfactory system, and Tisserand (Mills et al. 1995) proposed that *L. angustifolia* odors have an action similar to benzodiazepines in effecting gamma-aminobutyric acid (GABA) neurotransmission.

A study on dental patients who were exposed to lavender smells found that their anticipatory anxiety was much lower (Haag and Gould 1994). Hoferl et al. showed that linalool scents alone might counteract the psychological parameters brought on by stress. Linalool has been connected to several of the anxiolytic properties of lavender (Lantry et al. 1997).

Despite the fact that linalool and lavender oil have been shown to have good psychopharmacological effects in people, new research has found that linalool exposure can cause allergic reactions. Since 2003, the 7th Amendment to the European Cosmetic Directive has required that cosmetic products containing any of 26 natural ingredients, including linalool, be labeled as potentially allergenic. This is due to an increased awareness among European legislators of the allergenic properties of many common essential oil constituents (Ren and Gould 1994). While linalool itself might not be very allergenic, air exposure can cause it to auto-oxidize into a hyperoxide species (Hohmann et al. 1999), which can cause contact allergy reactions in mice (Heuberger et al. 2004). Using patch tests, it was discovered that 1.1% of 1511 dermatitis patients were sensitive to the linalool hyperoxide fraction and that 1.3% of those tested experienced allergic reactions to auto-oxidized linalool (Gamez et al. 1990). A follow-up investigation, again comprising 1511 dermatitis patients, revealed that 5–7% of test subjects experienced allergic irritation after being exposed to oxidized linalool at concentrations more than 6.0% (Perrucci et al. 1994).

**Table:** 

References	Test subjects	Number of volunteers	Compound	Dosage	Delivery	Method of assessment	Outcome
Firoozeei et al. 2021)	Elderly hospitalized for acute care	31	Lavender oil	“One drop” on “a pillow”	Olfactory	Observation	Enhanced sleep
Mameneh et al. 2021)	Healthy infants	30	Lavender oil	Data not shown/aromatic bath oil	Olfactory, trans dermal	Observation, salivary cortisol levels	Enhanced sleep, decreased stress
Fismer and Pilkington 2012)	Healthy adults	31	Lavender oil	Aromatic exposure in 2 min intervals	Olfactory	Polysomnographic recording	Enhanced sleep
Boelens 1995)	Healthy adult dental patients	343	Lavender oil	5 Drops of oil in10 mL diffused by candle	Olfactory	Modified dental anxiety scale, state trait anxiety inventory	Decreased anxiety
Smigielski et al. 2009)	Healthy adults	12 and 24	*R*-( −)- and *S*- ( +)-linalool	20 µL of various oil dilutions (between 0.003 and 30% of air)	Olfactory	Survey, electroencephalographic activity	Increased favorable impressions
Góra et al. 2005)	Healthy adults	24	*R*-( −)- and *S*- ( +)-linalool	2.7 mg/m^3^ (*R*-( −) linalool) and 9.8 mg/m^3^ (*S*-( +)-linalool) of air in room	Olfactory	Autonomic and endocrine system parameters including salivary cortisol levels	Decreased anxiety
Lis-Balchin and Hart 1999)	Dermatitis patients	1511	Linalool, myrcene, and caryophyllene, and oxidation products	0.5–3.9% of oxidized terpenoids, 20% non-oxidized linalool in petrolatum	Transdermal patch test	Observation of skin irritation	Contact allergy to terpenoid oxidation products
Mills et al. 1995)	Dermatitis patients	1511	Linalool, oxidized linalool	2–11% Petrolatum (0.80–4.4 mg/cm^2^)	Transdermal l patch test	Observation according to the International Contact Dermatitis Research Group guidelines	Contact allergy to oxidized linalool
Haag and Gould 1994)	Elderly hospitalized for dementia	21	Lavender oil	Data not shown	Olfactory, trans dermal	Observation of motor behaviors	Decreased agitation
Lantry et al. 1997)	Elderly hospitalized for dementia	15	Lavender oil	2% of air	Olfactory	Pittsburgh agitation scale	Decreased agitation
Clegg et al. 1982)	Elderly hospitalized for dementia	36	Lavender oil	3.5% of aqueous solution	Trans dermal	Mini-mental state examination	Increased cognition
Hohmann et al. 1999)	Elderly hospitalized in ICU short term	122	Lavender oil	1.0% of aqueous solution	Olfactory, trans dermal	Behavioral observation, blood pressure, heart rate, breath rate	Increased sedation
Perrucci et al. 1994)	Healthy adult females	96	Lavender oil	Cotton wood soaked with three drops of oil in a jar	Olfactory	Galvanic skin response	Increased relaxation
O’Brien 1999)	Healthy infants	45	Lavender oil	10% v/v	Olfactory	Electroencephalographic activity	Increased positive affect
Akhondzadeh et al. 2003)	Healthy adults	40	Lavender oil	10% v/v	Olfactory	Electroencephalographic activity	Increased positive mood, sedation
Kashani et al. 2011)	Healthy adult males	30	Lavender oil	“Four oil drops diluted with 20 mL hot water”	Olfactory	Coronary flow velocity reserve	Increased relaxation, coronary circulation
Mayaud et al. 2008)	Adult male	1	Lavender oil	2% in peanut oil	Trans dermal	Gas chromatography analysis of blood	Rapid accumulation (peak 20 min) and expulsion (90 min)
Hanamanthagouda et al. 2010)	Healthy adults	4	1,8-Cineole	Air passing over 4 mL for 20 min	Olfactory	Gas chromatography analysis of blood	Accumulation (peak _~_ 18 min) and expulsion half-life (104.6 min) of 1,8 cineole

The propensity of lavender essential oils to change the behavior of individuals with dementia has been evaluated in a large number of clinical investigations. It has been demonstrated that even inhaling lavender oils can lessen agitation in dementia patients (O’Brien 1999). Exposure to lavender aromatics has been proven to significantly reduce excessive motor behavior in people with dementia when combined with massage therapy (Akhondzadeh et al. 2003). Following dermal treatments (such as skin creams containing lavender oil), dementia patients demonstrated significantly lessened cognitive impairment throughout the course of a 4-week study (Kashani et al. 2011).

## Toxicology/precautions/contraindications allergy

Following topical application of lavender, some cases of hypersensitivity have been reported (Schaller and Korting 1995; Guillemain et al. xxxx). Both animals (Buchbauer et al. 1991; Rademaker 1994) and humans (Gabbrielli et al. 1988) have been proven to exhibit narcotic effects from lavender. It is also noteworthy that caution should be exercised in patients with known allergy/hypersensitivity to lavender. People with lavender allergies may develop modest local skin reactions when lavender oil is administered topically to the skin (Benito et al. 1996; Schaller and Korting 1995).

## Adverse effects

General: At the recommended dose, lavender is considered to be well tolerated with few side effects (Dunn et al. 1995; Motomura et al. 1999; Nagai et al. 2000). Dermatology: Case reports of mild dermatitis after topical application of lavender oil have been reported (Varma et al. 2000). After applying lavender oil to the pillows, the individual may develop itchy dermatitis on his face (Benito et al. 1996). The outbreak of lavender allergy was finally confirmed by a patch test. Changes in photosensitization and skin pigmentation have been reported after topical application of lavender oil (Schaller and Korting 1995; Ripple et al. 1998). Aromatherapy may be associated with central nervous system depression (Atanassova-Shopova and Roussinov 1970) and has been shown to have additive narcotic effects in rats when combined with barbiturates or chloral hydrates (Rademaker 1994; Ziegler 1996). Various chemotherapies have been linked to reversible neutropenia (Atanassova-Shopova and Roussinov 1970), as it has high oral dosage of perillyl alcohol (POH), a monoterpene component of lavender. Nausea, vomiting, and lack of appetite have been linked to high dosages of lavender (> 5.0 g/day) and large doses of the lavender component perillyl alcohol (POH) (Zweibel et al. 1997; Guillemain et al. 1989).

### Interactions

#### Lavender/drug interactions

Taking lavender and pentobarbital or chloral hydrate together significantly increases rat sleep time and narcotic effects (Ren and Gould 1994; Ziegler 1996). Combined use with other sedatives and hypnotics may have cumulative or synergistic effects. Lavender contains coumarin, which has the potential to enhance the effectiveness of anticoagulant treatments: anticoagulants, NSAIDs, and antiplatelet drugs. Lavender may increase GABA levels and enhance the sedative effects of GABA-based antiepileptic drugs [193]. The HMGCoA enzyme is inhibited by the cyclic monoterpenes contained in lavender, which lowers cholesterol levels in rats. The lavender component perillyl alcohol (POH) has been shown to inhibit the conversion of latte sterols to cholesterol.

#### Lavender/herb/supplement interactions

Sedating agents: In animal models, lavender has been shown to have sedative effect and to interact synergistically with sedatives such as pentobarbital and chloral hydrate (Ren and Gould 1994; Ziegler 1996). It can enhance the effects of many sedatives, such as kava and valerian root. Anticoagulant herbs/supplements: Coumarin, found in various concentrations in lavender, can help anticoagulants work more effectively.

#### Lavender/food interactions

Insufficient available evidence. Lavender/lab interaction low-density lipoprotein (LDL), total cholesterol, high-density lipoprotein (HDL): Orally administered lavender acts like an HMGCoA reductase inhibitor and raises HDL, according to animal experiments. However, it may lower total cholesterol/LDL [193].

## Conclusion

**Fig. 2:**
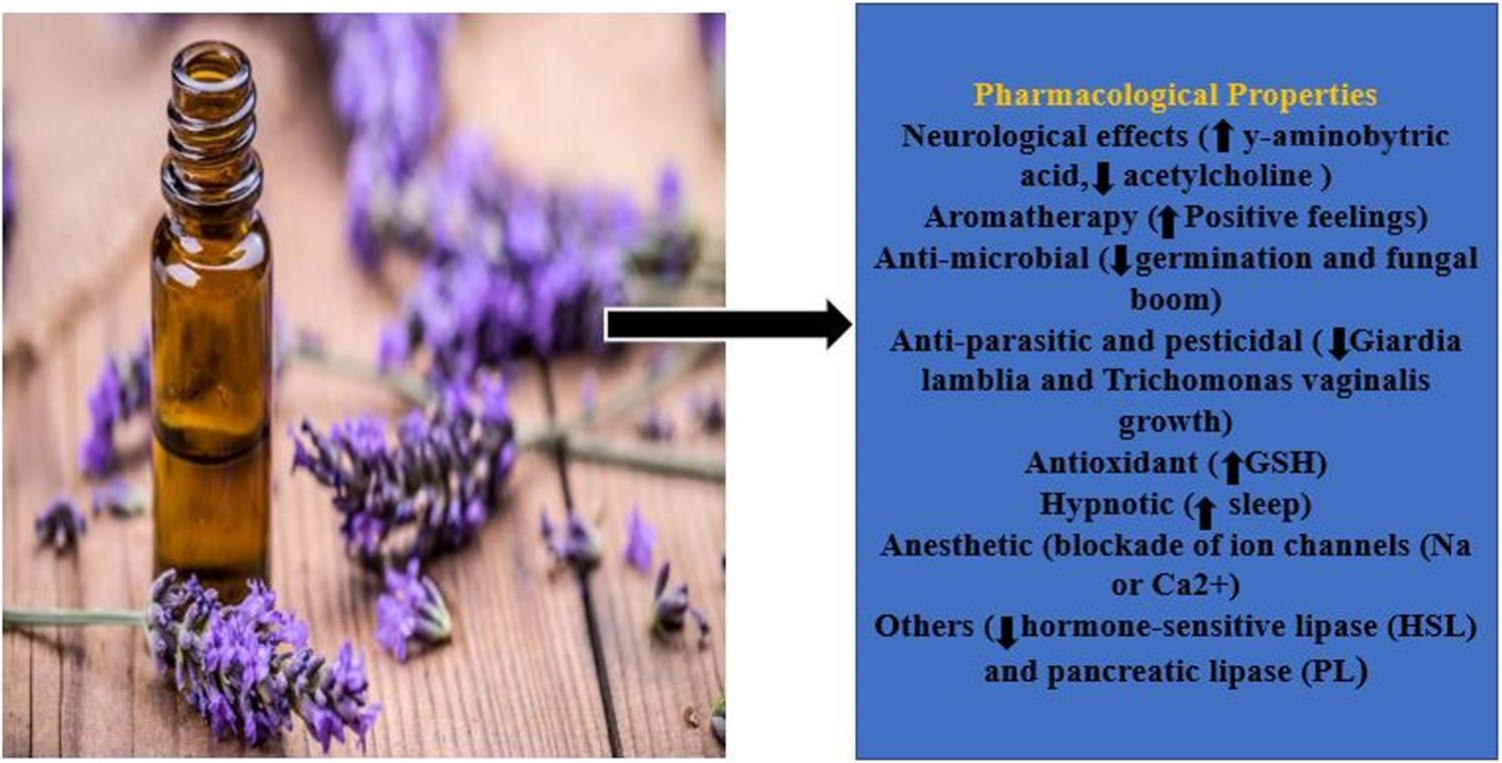
Mechanism of action of *Lavandula* sp. with emphasis on lavender oil

Lavender is a perennial evergreen plant that is also known as therapeutic or common lavender. It has antifungal, antibacterial, neurologic, antimicrobial, anti-parasitic, anti-diabetic, and analgesic effects (Fig. 2). It is widely used in the fields of cosmetics, perfumes, foods, and aromatherapy. Major bioactive components include essential oils, anthocyanins, phytosterols, sugars, minerals, coumaric acid, glycolic acid, valeric acid, ursolic acid, herniarins, coumarins, and tannins. *Lavandula* species has prospects for various biological applications especially its antioxidant activity. The antioxidant activity of lavender has been suggested to also contribute to wound healing. Advancement in drug development would enable characterization of various bioactive constituents. In vitro and in vivo studies on synergistic effect of lavender bioactive components with other molecules may prove to be a successful alternative to treating diseases associated with reduced immunity and oxidative stress.

## Data Availability

Data sharing is not applicable to this article as no datasets were generated or analyzed during the current study.
